# Clinical Value of Using Heart Rate Variability Biofeedback Before Elective CT Coronary Angiography to Reduce Heart Rate and the Need for Beta-Blockers

**DOI:** 10.1007/s10484-023-09590-6

**Published:** 2023-06-21

**Authors:** Patrick Langguth, Carmen Wolf, Sam Sedaghat, Monika Huhndorf, Johanne Frank, Marcus Both, Olav Jansen, Mona Salehi Ravesh, Annett Lebenatus

**Affiliations:** 1 Department for Radiology and Neuroradiology, University Hospital Schleswig-Holstein, Campus Kiel, Kiel, Germany; 2 Department of Diagnostic and Interventional Radiology, University Hospital Heidelberg, Heidelberg, Germany; 3 Department for Internal Medicine III, Molecular Cardiology and Angiology, University Hospital Schleswig-Holstein, Campus Kiel, Kiel, Germany

**Keywords:** Biofeedback, Computed tomography coronary angiography, Heart rate variability, Beta-blocker

## Abstract

The value of biofeedback before elective coronary computed tomography angiography (CCTA) to reduce patients’ heart rates (HR) was investigated in the current work. Sixty patients who received CCTA to exclude coronary artery disease were included in our study and separated into two groups: with biofeedback (W-BF) and without biofeedback (WO-BF). The W-BF group used a biofeedback device for 15 min before CCTA. HR was determined in each patient at four measurement time points (MTP): during the pre-examination interview (MTP1), positioning on the CT patient table before CCTA (MTP2), during CCTA image acquisition (MTP3), and after completing CCTA (MTP4). If necessary, beta-blockers were administered in both groups after MTP2 until a HR of less than 65 bpm was achieved. Two board-certified radiologists subsequently assessed the image quality and analyzed the findings. Overall, the need for beta-blockers was significantly lower in patients in the W-BF group than the WO-BF group (p = 0.032). In patients with a HR of 81–90, beta-blockers were not required in four of six cases in the W-BF group, whereas in the WO-BF group all patients needed beta-blockers (p = 0.03). The amount of HR reduction between MTP1 and MTP2 was significantly higher in the W-BF compared to the WO-BF group (p = 0.028). There was no significant difference between the W-BF and WO-BF groups regarding image quality (p = 0.179). By using biofeedback prior to elective CCTA, beta-blocker use could be decreased without compromising CT image quality and analysis, especially in patients with an initial HR of 81–90 bpm.

**Supplementary Information:**

The online version contains supplementary material available at 10.1007/s10484-023-09590-6.

## Introduction

Coronary artery disease (CAD) is one of the major cardiovascular diseases and the leading cause of morbidity and mortality worldwide (Dalen et al. 2014). In CAD, the formation of atherosclerotic plaque in the vessel wall in particular results in increasing stenosis of the coronary arteries. As the disease progresses, CAD is characterized by a mismatch of oxygen supply and demand by the heart and can potentially cause life-threatening complications such as myocardial infarction (Nakahara et al. 2017). Early diagnosis is therefore crucial.

Coronary computed tomography angiography (CCTA) is a contrast-enhanced technique for visualizing the coronary arteries. CCTA is minimally invasive, in contrast to cardiac catheterization. It is performed primarily in patients in whom the pretest probability (PTP) of CAD is low to intermediate (Knuuti et al. 2020). In prospective, electrocardiogram (ECG)-triggered CCTA, the heart rate (HR) of the patient being examined should ideally be regular and below 65 bpm, in order to achieve an image quality suitable for diagnosis and also to select CCTA protocols with reduced radiation doses (Earls 2009; , Sabarudin et al. 2013). Therefore, an ECG is performed as a standard part of CCTA examination to assess the HR and rhythm. If the HR is above 65 bpm, a cardioselective beta-blocker is often intravenously administered to reduce the patient’s HR to the target range (Sabarudin and Sun 2013). However, care must be taken before administering beta-blockers because these drugs can potentially lead to life-threatening bronchoconstriction, e.g., in patients with severe bronchial asthma (López-Sendón 2004). In addition, adverse cardiovascular side effects such as bradycardia, atrioventricular blockade, and heart failure may develop in some cases (Sabarudin and Sun 2013). Therefore, it is desirable to reduce HR without administering beta-blockers, without compromising imaging quality and analysis. The use of a biofeedback device could represent an appropriate noninvasive method to effectively lower HR while performing CCTA (Frank et al. 2010).

Changes in human biosignals (e.g., heart rate variability, HRV) can be measured and evaluated using a biofeedback device and presented to the user, for example, in visual form (Frank et al. 2010). For this purpose, an optical sensor can be used to determine the patient’s heartbeat and HRV. HRV is defined by the ability of an organism to adapt the HR. A high HRV indicates an organism’s ability to regulate the HR (Olshansky et al. 2008) and enables a healthy organism to adapt the HR to current needs (Shaffer and Ginsberg 2017). Physiologically, HR increases under physical strain or in stressful conditions and decreases again in relaxed situations (Kim et al. 2018; , Lehrer et al. 2020). HRV biofeedback is known to be able to improve HRV and adaptability of HR, but also to reduce anxiety and subjective stress levels in a short-term context (Goessl et al. 2017), which is crucial for returning HR to a resting level.

The aim of this prospective study was to evaluate the clinical value of using a biofeedback device in patients receiving elective CCTA to exclude CAD, in order to avoid the need for beta-blockers to further reduce HR, without compromising quality and analysis of the CT images.

## Methods

Our prospective and single-center study was conducted according to the declaration of Helsinki and was approved by our local institutional ethics board (No. D 549/19). All patients gave their informed consent in written form prior to inclusion.

### Patients

From December 2019 to January 2022, a total of 60 patients receiving elective CCTA to exclude CAD in our department were included in our study and separated into two groups: with biofeedback (W-BF) and without biofeedback (WO-BF). Biofeedback was applied in 30 patients before CCTA (W-BF), and another 30 patients constituted the control group without the use of biofeedback (WO-BF). Group assignments were randomized before the pre-examination interview (before MTP 1).

### Study Population Data

In the W-BF group, 14 (46.7%) subjects were female with a mean age of 59.2 ± 12.9 years, and in the control group (WO-BF) 17 subjects were female (56.7%) with a mean age of 58 ± 11.8 years. All demographic data (age, weight, body mass index, and gender) and clinical characteristics of our patients are summarized in Table 1.

**Table 1:** Demographic and clinical data of our study population. Metric data are given as mean and standard deviation; p-values were calculated using Students-t-test. For nominal data, absolute and relative frequencies are shown

	W-BF (n = 30)	WO-BF (n = 30)	p-value	
Demographic data				
Female gender, n (%)	14 (46.7)	13 (43.3)		
Age (years)	59.2 [12.9]	58.0 [11.8]	0.716	
Weight (kg)	81.3 [17.8]	83.7 [20.3]	0.636	
Height (cm)	174 [9.8]	172 [8.9]	0.316	
BMI (kg/m²)	26.7 [4.6]	28.1 [5.3]	0.266	
Clinical data				
Thoracic pain, n (%)	10 (33.3)	12 (40)		
Dyspnea, n (%)	15 (50)	14 (46.7)		
Post-infection, n (%)	2 (6.7)	4 (13.3)		
Positive family history, n (%)	16 (53.3)	16 (53.3)		
Asthma/COPD, n (%)	2 (6.7)	3 (10)		
Hypertension, n (%)	15 (50)	17 (56.7)		
Permanent use of beta-blocker, n (%)	9 (30)	7 (23.3)		
Psychopharmaceuticals, n (%)	4 (13.3)	8 (26.7)		
Nicotine abuse, n (%)	9 (30)	7 (23.3)		
Performing sportive activities, n (%)	16 (53.3)	15 (50)		
Performing Yoga, biofeedback, or autogenic training, n (%)	5 (16.7)	3 (10)		
Employed person, n (%)	20 (66.7)	16 (53.3)		
Stressful activity before examination, n (%)	3 (10)	6 (20)		
Consumption of coffee, black tea, or chocolate before examination, n (%)	19 (63.3)	18 (60)		

### Patient Evaluation

Following CCTA, all patients evaluated their subjective perceptions or stress levels before and during the examination, satisfaction, and perceived hospital atmosphere. These issues were rated on a scale from 1 to 6.

### HRV Biofeedback

In our study, we used a commercially available and mobile biofeedback device (“Qiu”, BioSign GmbH, Ottenhofen, Germany), which is a medical device and complies with all requirements of the Medical Device Directive 93/42/EEC (Supplementary file 1).

The Qiu is ball-shaped and can be optimally placed in the palm of the hand. An optical pulse sensor on the Qiu measures HR at the fingertip or palm. The Qiu is also equipped with a large light that can display a color spectrum from green, to yellow and orange, to red, depending on the HRV detected. When HRV is high, the Qiu lights up green, indicating a low stress level. In depressed HRV, the color of the Qiu changes from yellow to red, indicating an increased stress level. An additional blue light-emitting diode (LED) display on the Qiu shows breathing rate and speed, which was set to 6–7 breaths per minute for the present study. Patients were encouraged by the LED display to breathe deeply and slowly to increase the HRV and receive a green light on Qiu. These exercises were performed for a duration of 15 min in the W-BF group before CCTA.

### Study Design, Procedure, and Physiological Recording

**Fig. 1:**
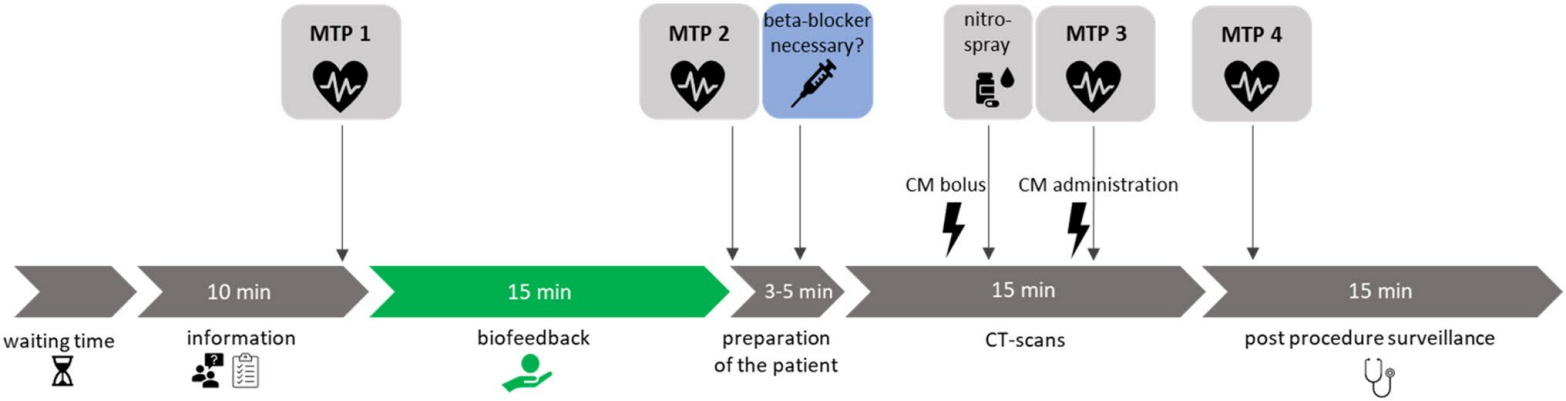
Timeline of the examination procedure. W-BF subjects performed biofeedback for 15 min between MTP 1 and MTP 2 (green). All other examination steps were the same for both groups (gray). *CM* contrast media. *MTP* measurement time point

The measurement timeline is shown in Fig. 1. Blood pressure and HR were assessed in both groups at four measurement time points (MTP):

MTP 1: During the pre-examination interview.

MTP 2: After the patient was positioned on the CT table before CCTA and potential beta-blocker administration.

MTP 3: During the CCTA image acquisition.

MTP 4: After completing the CCTA examination.

The patients in the W-BF group used the biofeedback device independently for 15 min between MTP 1 and MTP 2, whereas the WO-BF group waited during time without biofeedback. Otherwise, all examination steps were the same for the W-BF and WO-BF groups. If a beta-blocker was administered, the post-procedure surveillance time was prolonged to 30 min.

### CCTA Examination

All prospective, ECG-triggered CCTA examinations were performed using a 2 × 128-section multi-detector CT (SOMATOM Definition Flash, Siemens Healthcare GmbH, Forchheim, Germany) with the following acquisition parameters: 128 × 0.6 collimation, 0.28 s gantry rotation time, 120 kVp tube voltage, adapted mA tube current (range 35–800), and 1-mm-thick reconstructed images. An automated attenuation-based dose modulation (CARE Dose, Siemens Healthcare GmbH, Forchheim, Germany) was used in all patients. The total dose and injection rate of the iodine-based contrast medium (Imeron 350, Bracco, Milan, Italy) depended on the respective body mass index (BMI < 25: 5 ml/s, 65 ml; BMI 25–30: 5.5 ml/s, 70 ml; BMI > 30: 6 ml/s, 75 ml). CCTA datasets were acquired in all patients with a 4- to 6-s trigger delay while the bolus tracker was placed in the ascending aorta.

The breath-holding maneuver for subsequent CCTA image acquisition was briefly practiced with each patient after MTP 2. In patients with cardiac arrhythmia or/and with a HR above 65 bpm after this breath-holding maneuver, a beta-blocker (Metoprolol®) was administered gradually at a dose ranging from 1 to 15 mg until the target (desired) HR was achieved. Each patient received 0.8 mg nitrolingual spray 3–4 min before the CCTA examination.

### Image Reconstruction and Analysis

All examinations were analyzed in consensus by two board-certified radiologists (P. L. and A. L.), each with at least 7 years of experience interpreting cardiac CT images. For analysis of CCTA images, multiplanar and three-dimensional reconstructions were created. The commercially available, clinical software SyngoVia (Siemens Healthcare, Forchheim, Germany) was used to analyze the images, to evaluate the CAD-RADS (Coronary Artery Disease – Reporting and Data System), and to determine the Agatston Score (Janowitz et al. 1991). The image quality was graded according to a scoring scale of 1 to 4:

Grade 1: Good, without artifacts.

Grade 2: Moderate, some artifacts not influencing ability to make diagnosis.

Grade 3: Poor, artifacts influencing ability to make diagnosis, but still possible to make approximate assessment.

Grade 4: Nondiagnostic.

### Statistical Analysis

Normality was tested for all metric variables by using the Shapiro-Wilk test. If normality was given, data were reported as mean and standard deviation (SD); if not, median with interquartile are shown. To compare group values, we used the t-test for normally distributed data, whereas for data without normality and for ordinal data the Mann Whitney U-Test was performed. To analyze dependencies of nominal data we applied the X² test or Fisher’s exact test if the sample size was too small. Statistical significance for all tests was set at a level of p < 0.05. Statistical analysis was performed using the jamovi project, version 2.5.5 for Windows (Sydney, Australia, 2021).

## Results

### Overall Beta-Blocker Administration and HR Reduction

**Fig. 2:**
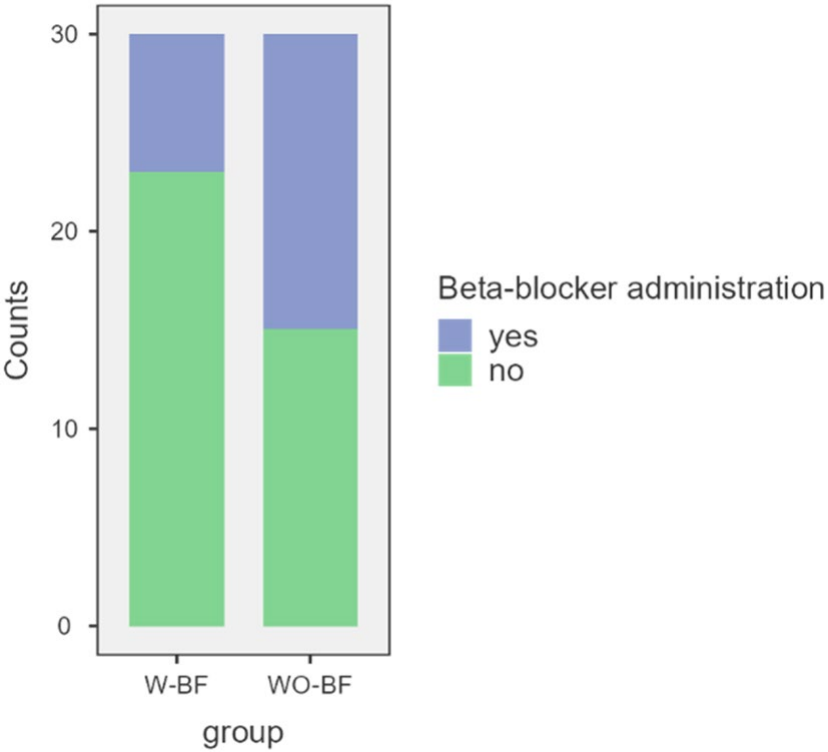
All patients in W-BF and WO-BF group with regard to beta-blocker administration

Overall, significantly fewer patients in the W-BF group [7/30 (23%)] required beta-blockers (p = 0.032) than in the WO-BF group [15/30 (50%); Fig. 2].

**Fig. 3:**
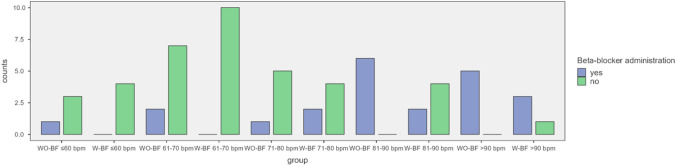
Comparison of administering beta-blockers between the W-BF and WO-BF groups, divided into different heart rate ranges

In patients with an initial HR of 71–80 bpm (WO-BF n = 6, W-BF n = 6), there was no significant difference in either group. In contrast, in four of six patients in the W-BF group with an initial HR of 81–90 bpm, beta-blockers were not required, whereas in the WO-BF group all patients needed beta-blockers (WO-BF n = 6, W-BF n = 6; p = 0.03). Almost all patients in both groups within a HR range of above 90 bpm (WO-BF n = 5, W-BF n = 4) required a beta-blocker, as illustrated in Fig. 3.

**Fig. 4:**
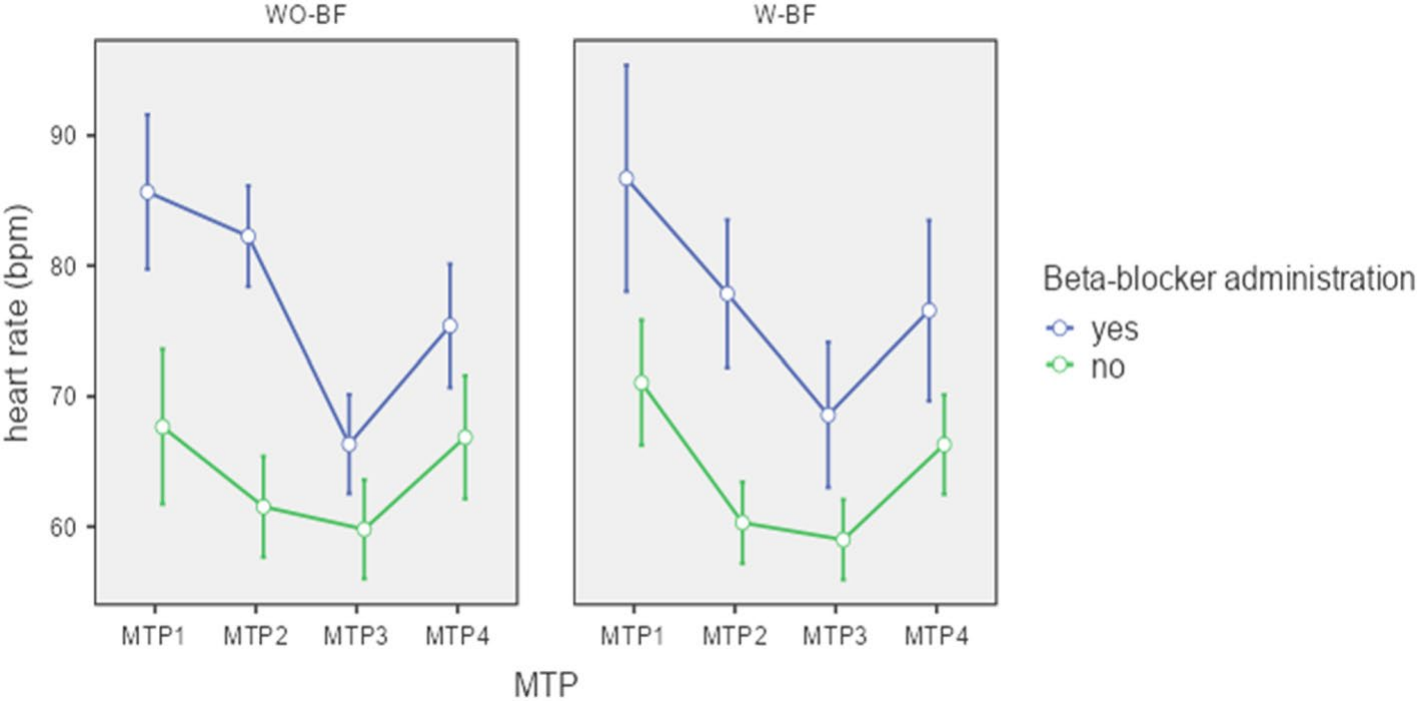
h in the course from MTP 1 to MTP 4 for W-BF and WO-BF subdivided in patients who received beta-blocker between MTP 2 and MTP 3 and those who did not

We found a significantly greater reduction in HR between MTP 1 and MTP 2 in the W-BF group compared to the WO-BF group (p = 0.024) with a HR-reduction of 9 [3.5;15.8] bpm in the W-BF and 4.77 ± 9.02 bpm in the WO-BF group. Figure 4 shows the different time course of HR values in the two groups, each subdivided in patients who received beta-blocker between MTP 2 und MTP 3 and patients who did not, illustrating the stronger drop of HR between MTP 1 and MTP 2 in the W-BF group. An overview of all HR values (MTP 1–4) of both groups is given in Supplementary file 2.

Unfortunately, all our patients in the WO-BF group with an initial HR of > 80 bpm (n = 11) received beta-blockers prior to the CCTA; thus, we were not able to compare patients with an HR > 80 bpm between the W-BF and WO-BF regarding the effect of biofeedback over the whole period, from admission (MTP 1) until the examination (MTP 3).

In the W-BF group, there was no significant difference in HR reduction between patients who were or were not diagnosed with CAD (p = 0.236). We found no significant differences between the W-BF and WO-BF groups in PTP of CAD (p = 0.5), in CAD-RADS grades (p = 0.556), and radiation dose to CCTA (p = 0.255), as shown in Supplementary file 3.

### Image Quality

There was no significant difference between the W-BF and WO-BF group regarding image quality (p = 0.179). Image quality was predominantly rated as good (Grade 1) in both groups, in 73.33% in the W-BF group and in 56.67% in the WO-BF group. Moderate image quality (Grade 2) was assessed in 23.33% in the W-BF and in 36.67% in the WO-BF group. Image quality was rated as poor (Grade 3) in only one patient (3.33%) in the W-BF group and in two patients (6.67%) in the WO-BF group. No CCTA examination in either group was classified as nondiagnostic (Grade 4). The results are summarized in Table 2.

**Table 2:** Analysis of image quality rated on a scale ranging from 1 (good) to 4 (nondiagnostic)

	W-BF (n = 30)	WO-BF (n = 30)	p-value
Image quality			0.179
1, n (%)	22 (73.33)	17 (56.67)	
2, n (%)	7 (23.33)	11 (36.67)	
3, n (%)	1 (3.33)	2 (6.67)	
4, n (%)	0 (0)	0 (0)	

Absolute and relative frequencies are shown for W-BF and WO-BF groups. P-value is calculated using Mann Whitney U-test

### Patient Evaluation

The evaluation did not reveal any significant differences in patient satisfaction and care (median grade of 1), waiting time, and premises (median grade of 2). The perceived hospital atmosphere was not significantly different between the two groups (p = 0.06), nor were the patients’ pretest stress level ratings significantly different between the two groups (p = 0.292). However, patients’ stress levels during the CCTA examination were rated lower in the W-BF than in the WO-BF group (p = 0.048). All analyzed data are summarized in Supplementary file 4.

## Discussion

Our study demonstrates the clinical value of using biofeedback before elective CCTA to reduce the use of beta-blockers in these patients, without compromising image quality and analysis.

The main findings of our study are that the use of biofeedback is an effective method for lowering HR during elective CCTA and is most effective at an initial heart rate of 81 to 90 bpm.

### Coronary Computed Tomography Angiography (CCTA)

The ESC guidelines were updated in 2019, particularly with regard to the pretest probability (PTP) for the prevalence of CAD (Knuuti et al. 2020). PTP clinically assesses whether CAD may be present, based on the patient’s symptoms, age, and gender, and is used to identify those patients requiring further evaluation (Bing et al. 2020). The PTP was significantly lower in newly studied patient groups in the 2019 guidelines than in those from 2013 and, in comparison, currently represents only around one third of those patients identified in the previous guidelines (Reeh et al. 2019). Due to the update of PTP, therefore, more patients have been in the PTP range with a recommendation for a non- or less-invasive diagnostic workup, such as functional MRI imaging or CCTA. The number of CCTA examinations has already increased recently and is expected to increase in the future (Reeves et al. 2021). CCTA provides an assessment of various aspects of coronary atherosclerosis: visualization of coronary artery narrowing, quantification of vascular plaque, or visualization of potential plaque instability (Schmermund et al. 2018).

Various factors can affect the image quality of a CCTA examination and thus the ability to evaluate the results. Here, the patient’s HR during CT image acquisition represents a major factor, which should ideally be under 65 bpm and be regular in order to provide artifact-free visualization of the coronary arteries and also to keep radiation exposure low. It would therefore be desirable to reduce the HR to the target range without having to administer beta-blockers to avoid the risk of the adverse effects of these drugs.

### HRV Biofeedback

Biofeedback has a positive impact on HRV and is beneficial for perceived stress and regulating emotions. Lin et al. demonstrated that HRV biofeedback had a positive effect on cardiac autonomy and thus hostility in patients with CAD (Lin et al. 2015). However, in most studies, participants completed multiple biofeedback sessions per week and over an extended period of time. In our study, patients were exposed to biofeedback for the first time and applied it for only 15 min. Nevertheless, we obtained significant benefits in the W-BF group as compared to the WO-BF group. We explain these advantages as being a result of a short-term reduction in stress levels due to the biofeedback through the known effect of regular breathing exercises, on the one hand (Li et al. 2018), and, on the other, by distraction, when patients are unaccustomed to the hospital atmosphere and thus can focus on nonemotional aspects in this setting. Saito et al. showed that there is an anxiety-reducing effect of HRV biofeedback already after the first application, independent of the patient’s ability to relax (Saito et al. 2021). We assume that there was a similar effect in our study population, which is also strengthened by the consistently positive evaluation regarding biofeedback in the W-BF group following the CCTA.

The similarly less strong reduction in HR in the control group (WO-BF) between MTP 1 und MTP 2 was probably due to the orthostatic regulation because the patients were in a sitting position at MTP 1 and in a lying position at MTP 2.

The best positive effect by biofeedback was found at a HR between 81 and 90 bpm at MTP 1. In this range, the W-BF group was superior to the control group (WO-BF) and achieved the greatest effect in reducing HR. However, we were not able to compare the effect of biofeedback on reducing HR in patients with a HR 81–90 from MTP 1 to MTP 3 due to a lack of patient data. Our data did show a significant reduction in HR in these patients from MTP 1 to MTP 2. In patients with a HR of > 90 bpm at MTP 1, HR was also reduced, but the reduction was not sufficient to completely prevent the need for beta-blockers. In five of 13 patients in the W-BF group who did not receive beta-blockers and had an initial HR of less than 80 (MTP 1), we observed an increase in HR during CCTA (MTP 3). In three of these patients, HR even increased above 65 bpm, which we attributed, on the one hand, to a reflexive response to the nitrolingual spray and, on the other, in response to the volume and the accompanying reaction, such as sensation of warmth, of the contrast administration. In none of the patients did this increase in HR limit image quality. Overall, we observed that patients in the W-BF group required comparatively less beta-blockers before reaching the target HR.

In our study, we did not find any demographic predictors of efficacy or factors for failure of biofeedback. This fact suggests that the applicability of biofeedback is probably independent of various demographic and clinical characteristics. However, the number of patients in our study was very small; therefore, much larger populations would have to be studied to draw further conclusions here.

In patients with CAD, low HRV values are described (Xhyheri et al. 2012). In our study, we could not detect any significant differences associated with the severity of the underlying CAD or its PTP in the W-BF and WO-BF groups. This fact could also be attributed to the small study population, though.

### Study Procedure (Applicability of Biofeedback Device in Clinical Routine)

A perceived disadvantage of using biofeedback in elective CCTA examinations is the additional time of 15 min and the need to initially explain the BF method during the pre-examination interview. In our clinical experience, patients often have to wait for a while before the examination can start; thus, the waiting time is not necessarily prolonged by performing biofeedback. After the pre-examination interview, we left the patients to themselves while using the biofeedback device; therefore, other than explaining the BF procedure, there was no limitation in work time and workload for the staff.

An advantage of successfully applying biofeedback and not using beta-blockers is that the recommended follow-up time of 30 min after intravenously administering beta-blockers is omitted and follow-up can be reduced to 15 min, due to contrast administration.

The visualization of the biosignal for the patients on the biofeedback device we used seemed to work well, but other forms of display, such as line graphs, could also be considered at this point.

### Image Analysis

In evaluating the CCTA images, no significant differences were observed between the two groups in terms of image quality and analysis.

### Limitations

Our study has some limitations. The small number of patients remains the main limitation of our study. Another point is that biofeedback was only performed until the patient was positioned on the CT table, before the CCTA examination. Although in most cases we found a sustained effect of HR reduction up to the CCTA, it is possible that we could not achieve the maximal effect of HRV biofeedback during the CCTA examination itself. This raises the question of whether HRV biofeedback could also be applied during the CCTA examination, for example, by integrating visual feedback into the CT scanner.

## Conclusion

Using biofeedback before elective CCTA examinations could offer a noninvasive and clinically valuable method to prevent the need for beta-blocker administration or reduce the administered dose of beta-blockers without compromising CT image quality and analysis. Biofeedback could be effective particularly in patients within an initial HR range of 81–90 bpm.

### Supplementary Information

Below is the link to the electronic supplementary material.

Supplementary material 1 (DOCX 93.9 kb)

## Abbreviations

ACS: Acute coronary syndrome
AP: Angina pectoris
BF: Biofeedback
BMI: Body mass index
bpm: Beats per minute
CA: Contrast agent
CAD: Coronary artery disease
CAD-RADS: Coronary artery disease—reporting and data system
CCTA: Coronary computed tomography angiography
CECT: Contrast-enhanced CT
CHD: Coronary heart disease
CM: Contrast media
CT: Computed tomography
ECG: Electrocardiogram
HR: Heart rate
HRV: Heart rate variability
ICA: Invasive coronary angiography
LAD: Left anterior descending artery
LCA: Left coronary artery
RCA: Right coronary artery
RCX: Right circumflex coronary artery
W-BF: With biofeedback
WO-BF: Without biofeedback
