# Correction to: Clinical Value of Using Heart Rate Variability Biofeedback Before Elective CT Coronary Angiography to Reduce Heart Rate and the Need for BetaBlockers

**DOI:** 10.1007/s10484-023-09596-0

**Published:** 2023-07-10

**Authors:** Patrick Langguth, Carmen Wolf, Sam Sedaghat, Monika Huhndorf, Johanne Frank, Marcus Both, Olav Jansen, Mona Salehi Ravesh, Annett Lebenatus

**Affiliations:** 1 Department for Radiology and Neuroradiology, University Hospital Schleswig-Holstein, Campus Kiel, Kiel, Germany; 2 Department of Diagnostic and Interventional Radiology, University Hospital Heidelberg, Heidelberg, Germany; 3 Department for Internal Medicine III, Molecular Cardiology and Angiology, University Hospital Schleswig-Holstein, Campus Kiel, Kiel, Germany

**Correction to: Applied Psychophysiology and Biofeedback**

10.1007/s10484-023-09590-6

The original version of this article unfortunately contained typo in all author names.

The given name and family name are swapped for all the authors and published incorrectly. Now, the correct author names are presented in this correction article.

The original article has been corrected.

