# Supplementary material for: Clinical Value of Using Heart Rate Variability Biofeedback Before Elective CT Coronary Angiography to Reduce Heart Rate and the Need for Beta-Blockers
Source: Appl Psychophysiol Biofeedback. 2023 Jun 21;48(4):393–401. doi: 10.1007/s10484-023-09590-6 (PMC10581922; doi:10.1007/s10484-023-09590-6)
Supplement: Supplementary file 1 — Supplementary material 1 (DOCX 93.9 kb) [file 10484_2023_9590_MOESM1_ESM.docx]

**Supplementary file 1:** Image of the biofeedback device "Qiu". The image was kindly provided by BioSign GmbH, Ottenhofen, Germany.


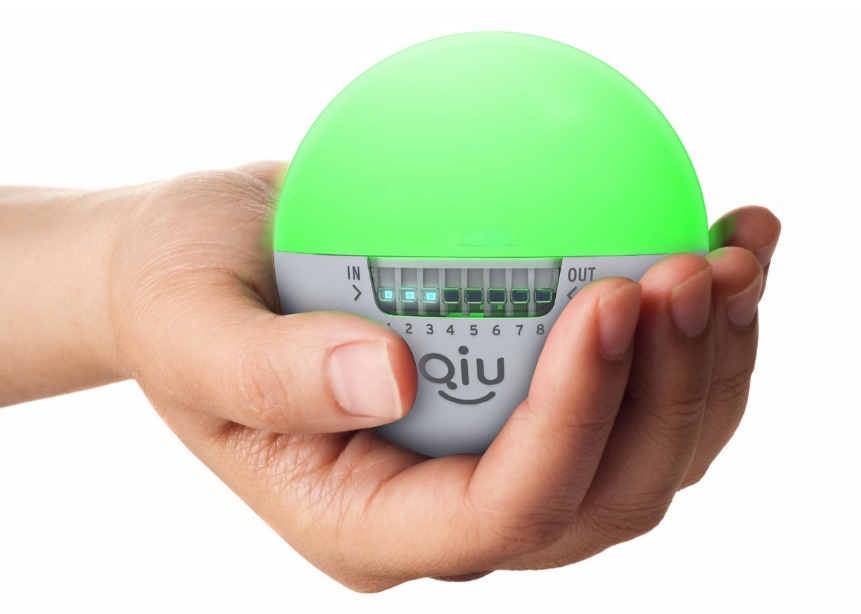


**Supplementary file 2:** Descriptive statistics of patients’ HR in bpm regarding MTP 1. If data are normally distributed, mean and standard deviation are given.

| HR in bpm | **W-BF** (n=30) | **WO-BF** (n=30) |
| --- | --- | --- |
| **MTP 1** | 74.7 ± 13.0 | 76.7 ± 17.7 |
| **MTP 2** | 62 [60;68.8] | 71.9 ± 13.8 |
| **MTP 3** | 61.2 ± 8.93 | 63.1 ± 7.25 |
| **MTP 4** | 68.7± 10.9 | 71.1 ± 9.03 |

**Supplementary file 3:** Absolute and relative frequencies of CAD-RADS grades are shown in the W-BF and WO-BF groups. Radiation dose and pretest probability are given as median and interquartile for both groups. P-values are tested using Mann Whitney U-Test.

|  | **W-BF** (n=30) | **WO-BF** (n=30) | p-value |
| --- | --- | --- | --- |
| **CAD-RADS** |  |  | 0.556 |
| 0, n (%) | 15 (50%) | 13(43.33%) |  |
| 1, n (%) | 1 (3.33%) | 12 (40%) |  |
| 2, n (%) | 3 (10%) | 1 (3.33%) |  |
| 3, n (%) | 4 (13,33%) | 1 (3.33%) |  |
| 4, n (%) | 7 (23.33%) | 0 (0%) |  |
| 5, n (%) | 0 (0%) | 3 (10%) |  |
|  |  |  |  |
| **Radiation dose** in mGy*cm | 139 [119;265] | 171 [135;271] | 0.255 |
| **Pretest probability** in % | 17 [6;21.3] | 13 [7;17] | 0.5 |
|  |  |  |  |

**Supplementary file 4:** Analysis of patients’ evaluations. Satisfaction was rated with grades ranging from 1 (very satisfied) to 6 (very dissatisfied). Stress level was rated with grades ranging from 1 (not excited) to 6 (extremely excited). For these parameters, median and interquartile are shown. P-values are calculated using Mann Whitney U-Test. Regarding patients’ statements concerning the biofeedback method, absolute and relative frequencies of agreement are given.

|  | **W-BF** (n=30) | **WO-BF** (n=30) | p-value |
| --- | --- | --- | --- |
| **Satisfaction with** |  |  |  |
| Service | 1 [1;2] | 1 [1;2] | 0.850 |
| Waiting time | 2 [1;2.75] | 2 [1;2] | 0.415 |
| Pre-examination interview | 1 [1;1] | 1 [1;1] | 1.0 |
| Care | 1 [1;1] | 1 [1;1] | 1.0 |
| Atmosphere | 1 [1;1] | 1 [1;2] | 0.060 |
| Premises | 2 [1;2] | 2 [1;2] | 0.348 |
| General impression | 1 [1;2] | 1 [1;2] | 0.886 |
|  |  |  |  |
| **Subjective excitement** |  |  |  |
| Before examination | 2 [2;3] | 2.5 [2;4] | 0.292 |
| During examination | 2 [2;2] | 2.5 [2;3] | 0.096 |
|  |  |  |  |
| **Statement about biofeedback method** |  |  |  |
| Biofeedback method was known, n (%) | 12 (40) | 5 (16.7) |  |
| Practicing biofeedback in daily life, n (%) | 3 (10) | 1 (3.3) |  |
| Assessing biofeedback as valuable, n (%) | 25 (83.3) | 10 (33.3) |  |
|  |  |  |  |
